# Clopidogrel combined with rivaroxaban in peripheral artery disease after revascularization

**DOI:** 10.3389/fphar.2024.1485380

**Published:** 2025-01-13

**Authors:** Min Lu, Jiaqi Li, Huanyu Ni, Tong Qiao, Baoyan Wang

**Affiliations:** ^1^ Changshu No.2 People’s Hospital, Affiliated Changshu Hospital of Nantong University, Changshu, Jiangsu, China; ^2^ Nanjing Drum Tower Hospital, Affiliated Hospital of Medical School, Nanjing University, Nanjing, Jiangsu, China

**Keywords:** peripheral artery disease, clopidogrel, rivaroxaban, efficacy, safety

## Abstract

**Background:**

To evaluate the efficacy and safety of clopidogrel-rivaroxaban combination compared to aspirin-rivaroxaban combination in patients with symptomatic peripheral artery disease (PAD).

**Methods:**

Consecutive patients with symptomatic PAD patients were analyzed from January, 2018 to June, 2022 at Nanjing Drum Tower Hospital. Patients were divided into two groups based on the antithrombotic therapy. The primary efficacy outcome was a composite of major adverse cardiovascular events (MACE) and major adverse limb events (MALE), and the primary safety outcome was major bleeding. Patients were followed until the first occurrence of any outcomes or the study end date (30 June 2024).

**Results:**

A total of 695 patients were enrolled into this study. The clopidogrel-rivaroxaban combination significantly reduced the risk of composite outcome (HR: 0.59, 95%CI: 0.41–0.83) without increasing the risk of major bleeding (HR: 0.68, 95%CI: 0.27–1.69). When analyzed separately, clopidogrel-rivaroxaban combination was associated with a reduced risk of MALE (HR: 0.61, 95%CI: 0.41–0.91), although no significant differences were observed in terms of MACE (HR: 0.64, 95%CI: 0.34–1.20) or all bleeding events (HR: 1.00, 95%CI: 0.52–1.93). In the subgroup analysis, there were no significant interactions between the treatment groups and the subgroups of age, diabetes, lesion sites, Rutherford classifications and renal function for composite outcome, MACE and MALE.

**Conclusion:**

The clopidogrel-rivaroxaban combination in PAD patients may offer enhanced cardiovascular protection without increasing the risk of bleeding complications. These findings suggested that clopidogrel could be a superior alternative to aspirin in dual antithrombotic therapy for PAD management.

## 1 Introduction

Peripheral artery disease (PAD) is a chronic atherosclerotic condition predominantly affecting the arteries in the lower extremities, leading to compromised blood flow and subsequent ischemic symptoms (Bierowski et al., 2023). PAD poses a significant public health challenge, particularly among elderly populations with combined conditions such as diabetes, hypertension and hyperlipidemia. This disease not only significantly impairs health-related quality of life but also heightens the risk of major adverse cardiovascular events (MACE) and major adverse limb events (MALE), such as amputation (Shishehbor and Castro-Dominguez, 2022). Effective management of PAD is essential to prevent these severe complications and improve patient outcomes.

Recent randomized controlled trials (RCTs) have demonstrated that the combination therapy with aspirin and rivaroxaban significantly reduced the incidence of MACE and MALE in patients with PAD (Eikelboom et al., 2017; Bonaca et al., 2020). This dual antithrombotic strategy offered enhanced vascular protection compared to monotherapy and has become a new therapeutic option for PAD management. However, despite the proven benefits of aspirin combined with rivaroxaban, some patients were unable to tolerate aspirin due to contraindications or adverse effects. Aspirin intolerance could arise from various conditions, including a history of gastrointestinal bleeding, peptic ulcer disease, aspirin-exacerbated respiratory disease or hypersensitivity reactions (Galli et al., 2024). In such cases, alternative antiplatelet agents such as clopidogrel were critical options. Clopidogrel, a P2Y12 inhibitor, is widely utilized in patients who cannot tolerate aspirin, providing a comparable antiplatelet effect. Current guidelines recommend low-dose aspirin or clopidogrel in patients with symptomatic PAD for improving cardiovascular prognosis (Twine et al., 2023; Aboyans et al., 2021).

Despite the established use of clopidogrel as alternatives to aspirin, the efficacy and safety in combination with rivaroxaban have not been extensively studied. Understanding the outcomes associated with different combinations of antiplatelet agents and rivaroxaban is crucial for optimizing treatment strategies. This retrospective cohort study aimed to evaluate the safety and efficacy of clopidogrel combined with rivaroxaban, offering evidence-based recommendations for antithrombotic therapy in PAD patients who cannot tolerate aspirin.

## 2 Materials and methods

### 2.1 Study design and setting

This study was a retrospective cohort analysis conducted at Nanjing Drum Tower Hospital, focusing on patients diagnosed with peripheral artery disease treated between 1 January 2018 and 30 June 2022. The study was approved by the Ethics Committee of Nanjing Drum Tower Hospital (Approval Number: 2021–198–03), and patient consent was waived due to the retrospective nature of the study.

Patients were diagnosed with symptomatic lower extremity atherosclerotic PAD and underwent revascularization as evidenced by following criteria (Aboyans et al., 2018): (1) clinically, by the presence of intermittent claudication, ischemic rest pain, ischemic ulceration or gangrene; (2) anatomically, by evidence of vascular occlusions revealed by computed tomography angiography or digital subtraction angiography; and (3) hemodynamically, by abnormal value of the ankle-brachial index.

Exclusion criteria included the following: (1) liver insufficiency (Child-Pugh class B or C) or renal insufficiency [creatinine clearance (CrCl) < 15 mL/min]; (2) single or dual antiplatelet therapy after revascularization; (3) autoimmune diseases or other diseases with symptoms similar to lower extremity PAD, including intermittent claudication, ulceration or necrosis (e.g., arteritis); (4) any clinical condition requiring systemic anticoagulation (e.g., atrial fibrillation).

Patients who underwent revascularization procedures were divided into two groups based on subsequent antithrombotic therapy: (1) aspirin plus rivaroxaban 2.5 mg/bid; (2) clopidogrel plus rivaroxaban 2.5 mg/bid. The index date was defined as the date of hospital admission. The follow-up period began when the patient received antithrombotic therapy after revascularization and continued until death or the end of the study period (30 June 2024), whichever occurred first. Patients were contacted via telephone for incomplete follow-up information.

### 2.2 Covariates

Data were extracted from electronic medical records, including patient demographics, comorbidities, renal function, medication history, lesion sites, Rutherford classifications, and revascularization history. CrCl was estimated using the Cockcroft-Gault formula, categorized into four grades: ≥80 mL/min, 50–79 mL/min, 30–49 mL/min and 15–29 mL/min. Patients were classified according to the Rutherford classification based on their symptoms (Aboyans et al., 2018): claudication (Grade I), ischemic rest pain (Grade II), and tissue loss (Grade III). Lesion sites were categorized based on their anatomical location into iliofemoral-popliteal occlusion, isolated calf occlusion, or long-segment occlusion. Revascularization strategies employed included both open surgical approaches, such as vascular and autovascular bypass grafting, and endovascular treatments, including percutaneous transluminal angioplasty, stent implantation, balloon dilation, drug-eluting stent implantation, drug-coated balloon dilatation and rotational atherectomy (Aboyans et al., 2018). Additionally, the concomitant use of medications such as statins, calcium channel blockers, angiotensin-converting enzyme inhibitors, and angiotensin receptor blockers was documented. All these variables were included in the analysis for inverse probability of treatment weighting (IPTW) adjustment.

### 2.3 Study outcomes

The primary efficacy outcome was a composite of MACE and MALE, with MACE defined as myocardial infarction, ischemic stroke, and death from cardiovascular causes, and MALE defined as urgent revascularization, acute limb ischemia and major amputation. The primary safety outcome was major bleeding as defined by the International Society on Thrombosis and Haemostasis (ISTH) (Schulman et al., 2005). Secondary clinical outcomes included all bleeding events, such as major bleeding and clinically relevant non-major (CRNM) bleeding, as well as MACE and MALE, each analyzed separately.

### 2.4 Statistical analysis

In this study, the incidence of missing data was only 0.5%. Specifically, records with missing values were excluded from the dataset. To adjust for potential confounding factors and ensure balanced baseline characteristics between the two groups, we employed propensity score-based IPTW. The propensity score for each patient was estimated using a multinomial logistic regression model that included variables such as patient demographics, combined diseases, renal function, medication history, lesion sites, Rutherford classifications and other relevant clinical characteristics. Inverse probability weights were calculated for each patient based on their propensity score. These weights were then applied to the regression models to create a weighted pseudo-population in which the distribution of baseline covariates was independent of the treatment assignment. After applying the IPTW, we assessed the balance of baseline characteristics between treatment groups by calculating standardized mean differences (SMD) before and after weighting, with an SMD ≤0.1 indicating a negligible difference between the groups. The weighted incidence rates per 100 person-years of clinical outcomes were calculated. The risk of clinical outcomes for the groups were evaluated using Kaplan-Meier survival analysis with the log-rank test or multivariate analysis using weighted Cox proportional hazard regression models with IPTW. The proportional hazards assumption was checked using the Schoenfeld residuals. The 95% confidence intervals (CI) for hazard ratios (HR) were calculated using the aspirin-rivaroxaban combination group as the reference.

## 3 Results

### 3.1 Patient characteristics

**FIGURE 1:**
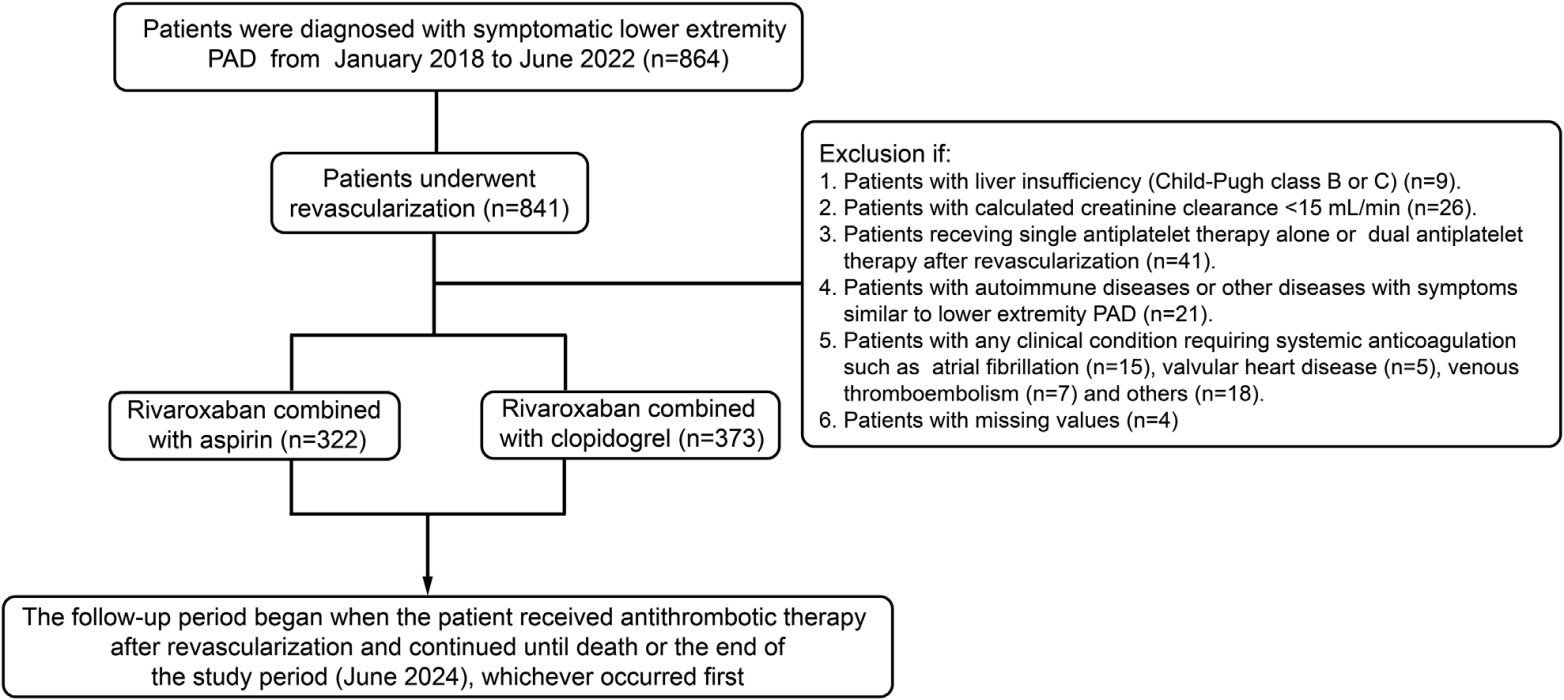
Flow diagram of patients included, excluded and analyzed in this study.

A total of 695 patients were included in this study (Figure 1). Baseline characteristics of the patients were presented in Table 1. Generally, patients receiving clopidogrel combined with rivaroxaban were older, had a higher proportion of angiotensin-converting enzyme inhibitors (ACEI)/angiotensin receptor blockers (ARB) and statin use before propensity score weighting. Conversely, patients receiving aspirin combined with rivaroxaban had a higher proportion of ischemic stroke, β-receptor blocker use and a history of previous revascularization. There were significant differences in the distribution of CrCl grades, lesion sites and revascularization strategy between the two groups. After IPTW, baseline characteristics were well balanced between the two groups.

**TABLE 1:** Baseline characteristics of patients receiving aspirin or clopidogrel before and after IPTW.

Items	Before IPTW			After IPTW		
	Aspirin group (n = 322)	Clopidogrel group (n = 373)	SMD	Aspirin group (n = 317)	Clopidogrel group (n = 378)	SMD
Female	84 (26.09%)	94 (25.20%)	0.020	81 (25.56%)	96 (25.40%)	0.001
Age (years)	71.04 ± 10.74	72.34 ± 11.73	0.116	71.42 ± 10.24	71.64 ± 12.55	0.019
BMI (kg/m^2^)	22.90 ± 3.66	22.65 ± 3.28	0.072	22.87 ± 13.59	22.84 ± 13.42	0.008
Rutherford classifications			0.055			0.005
Grade Ⅰ	109 (33.85%)	130 (34.85%)		108 (34.07%)	129 (34.13%)	
Grade Ⅱ	102 (31.68%)	124 (33.24%)		104 (32.81%)	124 (32.80%)	
Grade Ⅲ	111 (34.47%)	119 (31.90%)		105 (33.12%)	125 (33.07%)	
Combined diseases						
Hypertension	229 (71.12%)	268 (71.85%)	0.016	231 (72.87%)	270 (71.43%)	0.027
Diabetes	142 (44.10%)	153 (41.02%)	0.062	140 (44.16%)	165 (43.65%)	0.007
Coronary heart disease	51 (15.84%)	66 (17.69%)	0.050	51 (16.09%)	63 (16.67%)	0.018
Ischemic stroke	89 (27.64%)	84 (22.52%)	0.118	81 (25.55%)	94 (24.87%)	0.015
Medication use						
ACEI/ARB	70 (21.74%)	104 (27.88%)	0.143	79 (24.92%)	93 (24.60%)	0.006
β-receptor blocker	32 (9.94%)	22 (5.90%)	0.150	25.93 (8.17%)	32.16 (8.51%)	0.012
Calcium channel blocker	135 (41.93%)	161 (43.16%)	0.025	131 (41.32%)	154 (40.74%)	0.015
Statin	106 (32.92%)	252 (67.56%)	0.739	159 (50.16%)	190 (50.26%)	<0.001
Cilostazol	142 (44.10%)	153 (41.02%)	0.062	131 (41.32%)	159 (42.06%)	0.015
CrCl (mL/min)			0.272			0.038
≥80	101 (31.37%)	79 (21.18%)		84 (26.50%)	98 (25.93%)	
50–79	145 (45.03%)	210 (56.30%)		163 (51.42%)	194 (51.32%)	
30–49	61 (18.94%)	61 (16.35%)		55 (17.35%)	66 (17.46%)	
15–29	15 (4.66%)	23 (6.17%)		15 (4.73%)	20 (5.29%)	
Previous revascularization	50 (15.53%)	37 (9.92%)	0.169	41 (12.93%)	53 (14.02%)	0.028
Lesion sites			0.111			0.015
Iliofemoral-popliteal	122 (37.89)	125 (33.51)		115 (36.28%)	135 (35.72%)	
Isolated crural	33 (10.25)	34 (9.12)		32 (10.09%)	40 (10.58%)	
Long segment	167 (51.86)	214 (57.37)		170 (53.63%)	203 (53.70%)	
Revascularization strategy			0.102			0.040
Open surgery	55 (17.08%)	50 (13.40%)		60 (18.93%)	65 (17.20%)	
Endovascular treatment	267 (82.92%)	323 (86.60%)		257 (81.07%)	313 (82.80%)	

### 3.2 Clinical outcomes

**FIGURE 2:**
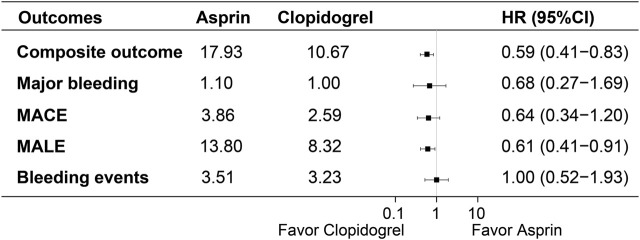
Weighted incidence rates, HR and 95% CI of clinical outcomes between the groups.

**FIGURE 3:**
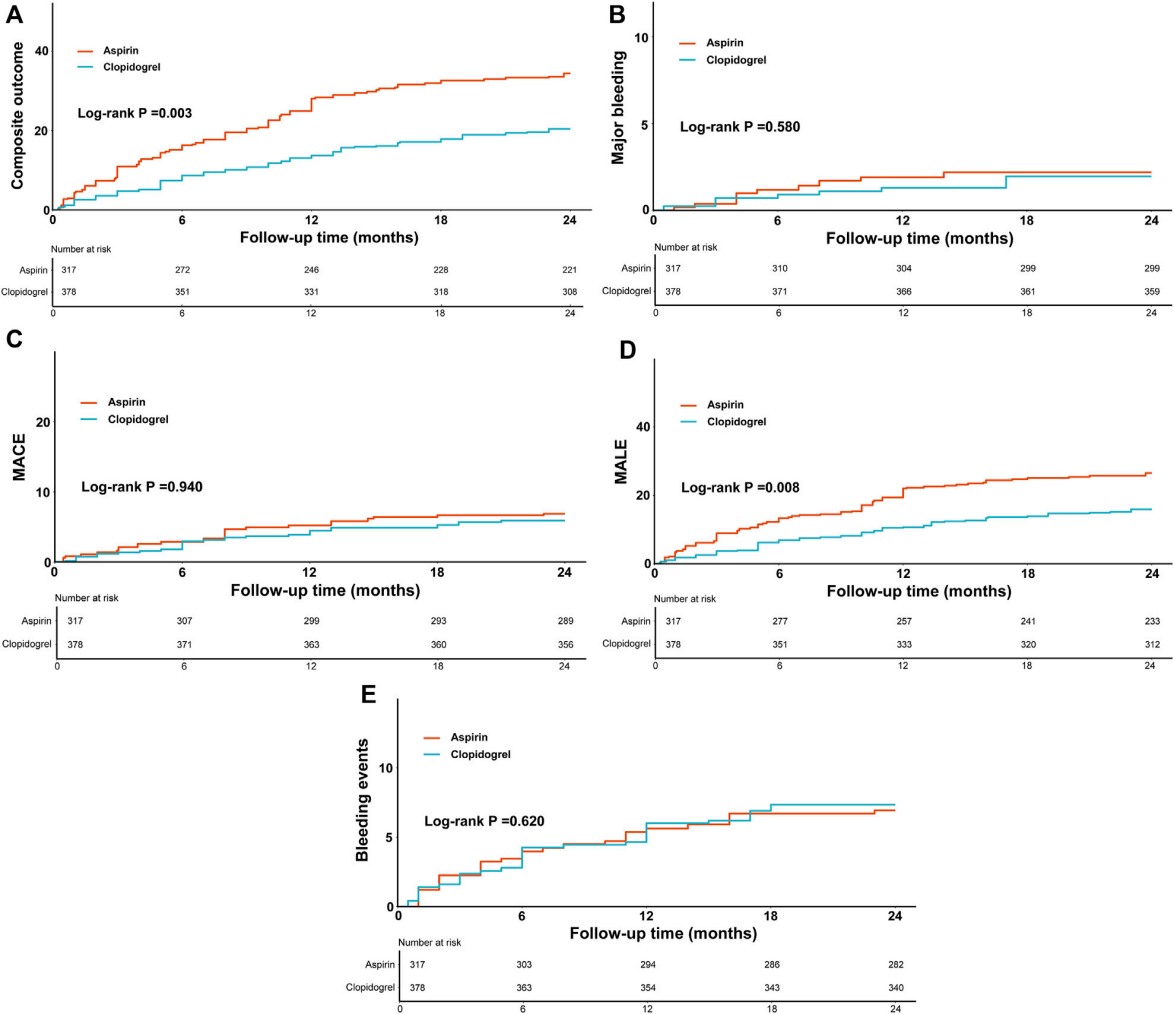
The weighted cumulative incidence curves of composite outcome **(A)**, major bleeding **(B)**, MACE **(C)**, MALE **(D)** and bleeding events **(E)** between the groups.

The actual incidence rates, weighted incidence rates per 100 person-years, HR values and 95%CI for the clinical outcomes between the two groups were presented in Supplementary Table SI and Figure 2. Notably, the majority of clinical outcomes occurred within the first year of follow-up, as shown in Figure 3, which indicated the early onset of adverse outcomes in patients with PAD. 92 patients in the aspirin group and 73 patients in the clopidogrel group experienced the composite outcome, resulting in a significant difference in the risk of these events between the two groups (HR: 0.59, 95% CI: 0.41–0.83). A further analysis of the clinical outcomes revealed that the incidence of MALE was significantly higher than that of MACE. Patients receiving clopidogrel-rivaroxaban combination had a lower risk of MALE (HR:0.61, 95%CI: 0.41–0.91), compared to those receiving aspirin-rivaroxaban combination. There was no significant difference in the risk of MACE, major bleeding and bleeding events between the groups.

### 3.3 Subgroup analysis

**FIGURE 4:**
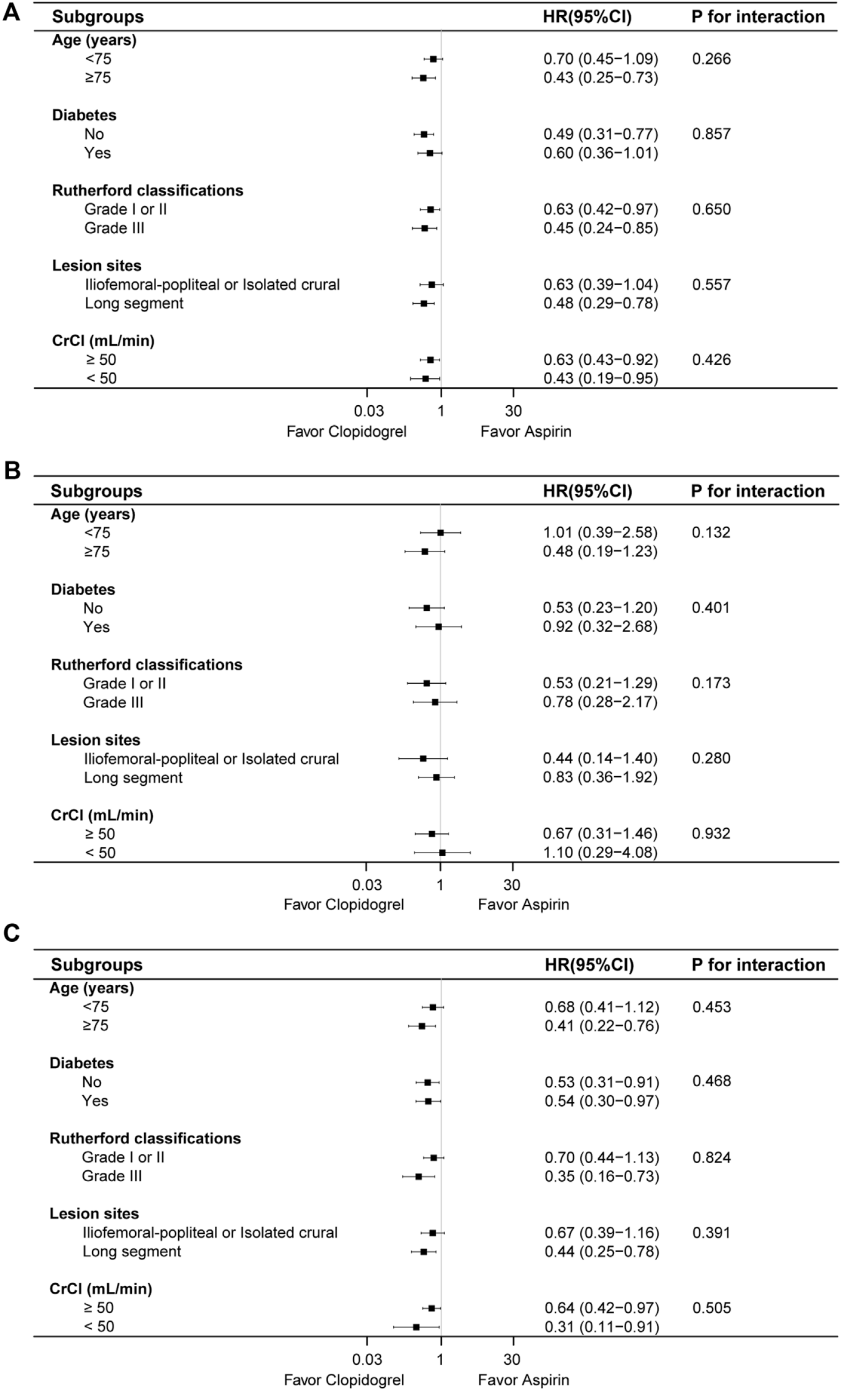
The risk of composite outcome **(A)**, MACE **(B)** and MALE **(C)** between the two groups based on various subgroups.

Subgroup analysis was performed based on age, diabetes, Rutherford classifications, lesion sites and renal function (Figure 4). In patients aged ≥75 years, clopidogrel group reduced the risk of composite outcome and MALE (HR: 0.43, 95% CI: 0.25–0.73; HR: 0.41, 95% CI: 0.22–0.76), similar in the lesion location subgroups. In patients without diabetes, clopidogrel group was associated with a lower incidence of composite outcome (HR: 0.49, 95% CI: 0.31–0.77) and MALE (HR: 0.53, 95% CI: 0.31–0.91), with a similar trend for patients with diabetes (HR: 0.54, 95% CI: 0.30–0.97). For Rutherford classifications, clopidogrel group reduced the risk of composite outcome across different grades (HR: 0.63, 95% CI: 0.42–0.97; HR: 0.45, 95% CI: 0.24–0.85), but no risk reduction was observed for MALE in grade I or II (HR: 0.70, 95% CI: 0.44–1.13). In terms of renal function, clopidogrel group consistently lowered the risk of composite outcome and MALE across renal strata. There were no statistical differences between the two groups for MACE in different subgroups. Overall, no significant interactions were found between treatment groups and subgroups (P for interaction >0.05).

## 4 Discussion

Antithrombotic therapy played a crucial role in the secondary prevention of patients with peripheral artery disease. However, the optimal antithrombotic strategy in terms of duration and intensity remained a topic of debate, particularly in the long term (Golledge, 2022). Even with standard antiplatelet and lipid-lowering therapies, patients with symptomatic PAD still experienced a high incidence of adverse cardiovascular events, indicating the need for more intensive antithrombotic therapy (Polonsky and McDermott, 2021).

In this study, compared with the aspirin-rivaroxaban combination, rivaroxaban combined with clopidogrel significantly reduced the risk of MACE and MALE composite outcome. Major bleeding was a critical concern with dual antithrombotic therapy. The VOYAGER PAD trial reported an increased risk of major bleeding with aspirin and rivaroxaban compared to aspirin alone (Bonaca et al., 2020). Our study found similar major bleeding rates between the clopidogrel-rivaroxaban combination and aspirin-rivaroxaban combination. However, the incidence of the composite outcome among patients in our study who received rivaroxaban combined with aspirin was significantly higher than the incidence of that observed in VOYAGER PAD trial. This may be attributed to the higher proportion of patients with severe symptoms in our study, such as rest pain or tissue damage. These patients typically presented with more advanced vascular wall lesions and an elevated risk of vessel occlusion. In this context, clopidogrel-rivaroxaban combination remained effective in reducing the risk of composite outcome in high-risk PAD patients, providing more effective protection against cardiovascular adverse events.

A systematic review and meta-analysis evaluated the effectiveness and safety of low-dose-rivaroxaban in patients with coronary artery disease and/or peripheral artery disease taking antiplatelets (Bucci et al., 2024). The results showed that low-dose rivaroxaban combined with any antiplatelet agent reduced the risk of cardiovascular events. When anticoagulation and antiplatelet therapies were used in combination, they may significantly reduce thrombosis formation through a synergistic effect, as demonstrated in current studies. In addition to platelets activation and aggregation, coagulation was another crucial factor in thrombosis formation (Kim et al., 2020; Meah et al., 2020). Both thrombin and factor Xa, through the protease-activated receptors (PARs), enhanced platelet activation with a continuous cross-talk between the coagulation and platelet pathway (Petzold et al., 2020). Previous studies have indicated an increased platelet activation during the acute phase of myocardial infarction, whereas thrombin generation appeared to persist for an extended duration in the post-acute coronary syndrome phase (Ohman et al., 2017). Based on this evidence, several studies have explored the impact of combination therapies involving the addition of anticoagulants to antiplatelet drugs on residual cardiovascular risk (Bonaca et al., 2020; Zannad et al., 2018). The COMPASS trial demonstrated the benefits of combining aspirin with rivaroxaban, showing a significant reduction in major adverse cardiovascular events and major adverse limb events compared to aspirin alone (Eikelboom et al., 2017).

Compared to aspirin, which primarily inhibited cyclooxygenase and platelet aggregation, clopidogrel operated via a different mechanism, potentially providing more robust antithrombotic protection. Clopidogrel monotherapy has been evaluated in patients at risk of ischemic events (CAPRIE) enrolling a broad population with stable atherosclerotic cardiovascular disease (CAPRIE Steering Committee, 1996). In a subgroup analysis of this study, clopidogrel demonstrated a MACE reduction in patients with symptomatic PAD when compared to aspirin (HR: 0.78, 95%CI: 0.65–0.93) and showed no significant difference in intracranial hemorrhage risk. Apart from patients with PAD, clopidogrel has also shown superiority over aspirin monotherapy for secondary prevention following percutaneous coronary intervention (PCI). The HOST-EXAM trial showed that clopidogrel monotherapy, compared to aspirin monotherapy during the chronic maintenance period after PCI significantly reduced the risk of future adverse clinical events (Koo et al., 2021). This observation may be explained by the prior studies that P2Y12 receptor inhibition was associated with a greater reduction of platelet function and thrombin formation than cyclooxygenase inhibition in moderate-to high-risk patients with coronary artery disease (Park et al., 2021).

Furthermore, subgroup analysis was performed based on the age, diabetes, renal function, Rutherford classifications and lesion sites. There was no statistical difference between the treatment groups in different subgroups for composite outcome. Clopidogrel combined with rivaroxaban could significantly reduce the risk of MALE in the patients aged ≥75 years old, Rutherford classification grade Ⅲ and long-segment lesion compared to the corresponding subgroups, however, there was no significant interaction. In the *post hoc* analysis of HOST-EXAM trial (Rhee et al., 2023; Lee et al., 2024), clopidogrel monotherapy demonstrated a lower rate of major adverse cardiac events compared to aspirin monotherapy in patients requiring antiplatelet monotherapy after PCI, regardless of the presence of diabetes or age. In our study, the results of the subgroup analysis were consistent with those of the overall population, indicating that the efficacy was uniform across different subgroups. This suggested that the clopidogrel-rivaroxaban combination was equally effective in diverse patient groups, thus enhancing the reliability and generalizability of the findings.

This study has certain limitations that should be addressed in future research. Firstly, this was a retrospective cohort study which may not account for all potential confounding variables that could influence the observed outcomes, even though the IPTW method was used to balance the covariate differences between the groups. Secondly, the sample size in the study was insufficient that may not adequately represent the population, affecting the credibility and validity of the study. However, the high incidence rate of clinical outcomes meant that significant associations or effects can be more readily observed, potentially making study conclusions more apparent.

## 5 Conclusion

This study indicated that clopidogrel-rivaroxaban combination significantly reduced the composite outcome of adverse cardiovascular and limb events in patients with PAD, compared to aspirin-rivaroxaban combination, without increasing the incidence of major bleeding. Therefore, clopidogrel-rivaroxaban combination was a superior alternative to aspirin in dual antithrombotic therapy, highlighting its potential therapeutic benefit in this patient population.

## Data Availability

The raw data supporting the conclusions of this article will be made available by the authors, without undue reservation.
