# Supplementary material for: Clopidogrel combined with rivaroxaban in peripheral artery disease after revascularization
Source: Front Pharmacol. 2025 Jan 13;15:1485380. doi: 10.3389/fphar.2024.1485380 (PMC11770050; doi:10.3389/fphar.2024.1485380)
Supplement: Supplementary file 1 [file Table1.pdf]

**Table I** The actual incidence rates, HR and 95% CI of clinical outcomes between the groups.

| Group | Composite outcome | Major bleeding | MACE | MALE | Bleeding events |
|---|---|---|---|---|---|
| Aspirin (n=322) | 92 (28.57%) | 8 (2.48%) | 24 (7.45%) | 73 (22.67%) | 21 (6.52%) |
| Clopidogrel (n=373) | 73 (19.57%) | 7 (1.88%) | 22 (5.90%) | 56 (15.01%) | 28 (7.51%) |
